# EndoViT: pretraining vision transformers on a large collection of endoscopic images

**DOI:** 10.1007/s11548-024-03091-5

**Published:** 2024-04-03

**Authors:** Dominik Batić, Felix Holm, Ege Özsoy, Tobias Czempiel, Nassir Navab

**Affiliations:** 1 Chair for Computer Aided Medical Procedures, Technical University Munich, Munich, Germany; 2 Carl Zeiss AG, Munich, Germany

**Keywords:** Endoscopy video analysis, Vision transformer, Pretraining

## Abstract

**Purpose:**

Automated endoscopy video analysis is essential for assisting surgeons during medical procedures, but it faces challenges due to complex surgical scenes and limited annotated data. Large-scale pretraining has shown great success in natural language processing and computer vision communities in recent years. These approaches reduce the need for annotated data, which is of great interest in the medical domain. In this work, we investigate endoscopy domain-specific self-supervised pretraining on large collections of data.

**Methods:**

To this end, we first collect Endo700k, the largest publicly available corpus of endoscopic images, extracted from nine public Minimally Invasive Surgery (MIS) datasets. Endo700k comprises more than 700,000 images. Next, we introduce EndoViT, an endoscopy-pretrained Vision Transformer (ViT), and evaluate it on a diverse set of surgical downstream tasks.

**Results:**

Our findings indicate that domain-specific pretraining with EndoViT yields notable advantages in complex downstream tasks. In the case of action triplet recognition, our approach outperforms ImageNet pretraining. In semantic segmentation, we surpass the state-of-the-art (SOTA) performance. These results demonstrate the effectiveness of our domain-specific pretraining approach in addressing the challenges of automated endoscopy video analysis.

**Conclusion:**

Our study contributes to the field of medical computer vision by showcasing the benefits of domain-specific large-scale self-supervised pretraining for vision transformers. We release both our code and pretrained models to facilitate further research in this direction: https://github.com/DominikBatic/EndoViT.

## Introduction

Minimally Invasive Surgery (MIS) is quickly becoming one of the most common styles of surgical procedures in the world [21]. In contrast to open surgery, MIS lowers the chance of infection and speeds up the recovery rate. As MIS procedures use endoscopic cameras, it has become possible to analyze large amounts of video data, leading to the development of surgical assistance systems. These systems can detect errors and provide decision support to improve patient outcomes [17]. Additionally, cataloging recorded surgical procedures provides valuable insights to surgeons, enabling them to learn and improve their techniques [23]. To achieve these goals, the community has thoroughly investigated the task of Surgical Phase Recognition [6, 26] and successfully managed to detect and localize surgical instruments [13]. Today, more challenging tasks are being explored, such as the newly introduced action triplet recognition [21]. It requires not only detecting surgical instruments, actions, and anatomies but also determining the relationship between them. Other works focus on the segmentation of tools and tissues[5], as well as multi-level learning, combining several tasks at once[27].

In general deep learning, the transformer [28] architecture has had a tremendous impact in recent years. Its success can be attributed to the introduction of self-supervised pretraining methods, such as Masked Language Modeling. The idea is straightforward: A percentage of input words are randomly masked out, and the model is tasked with predicting the missing input. Despite its simplicity, it presents a challenging self-supervised task. This approach has led to a paradigm shift in which a transformer network is first pretrained on large amounts of unlabeled data in order to create a model with a general understanding of the underlying domain. Later on, this model can be finetuned for a specific downstream task using significantly fewer annotations. With the advent of Vision Transformers (ViT) [8], similar strategies such as Masked Image Modeling have been developed for computer vision [2, 9, 29], showing equally high benefit in complex computer vision tasks.

Despite the advancements in computer vision and natural language processing, the progress of artificial intelligence methods in the medical field has been slower due to the insufficient amount of annotated data for developing data-driven approaches [27]. While the largest endoscopic dataset, Cholec80 [26], only contains 200k images, computer vision datasets can reach hundreds of millions of images [25]. Additionally, downstream medical tasks requiring complex annotations, such as pixel-wise segmentations, often have less than 10k images [11]. Pretraining models on larger datasets could be used to overcome this challenge. However, so far, only natural image datasets are generally available at the required size, which leaves a significant domain gap to endoscopic videos.

In this study, we use endoscopy domain-specific large-scale pretraining to bring advances from the computer vision community to the medical domain. Toward this objective, our contributions are threefold:

1. We compile the largest publicly available collection of unlabeled endoscopic data, Endo700k, consisting of more than 700,000 images.
2. We introduce the first publicly available endoscopy-pretrained vision transformer, EndoViT.
3. We analyze, through extensive experiments and ablation studies, the effect of endoscopy pretraining on the downstream tasks of surgical phase recognition, surgical action triplet recognition, and semantic segmentation.

## Methodology

### Dataset preparation

To enable effective endoscopy-specific pretraining, we have created the largest publicly available collection of raw endoscopic data, Endo700k. Endo700k is formed by combining nine publicly available MIS datasets comprising more than 700,000 images. An overview of the individual datasets is provided in Table 1. Endo700k contains a diverse set of endoscopic procedures, both manual and robot-assisted, with several surgery types such as prostatectomy, cholecystectomy, gastrectomy, proctocolectomy, rectal resection, and sigmoid resection. Furthermore, multiple different surgical actions, anatomies, and many surgical instruments, which are present in different shapes and sizes, are included. The downstream evaluation experiments are conducted on the Cholec80 dataset [26] and its subvariants CholecT45 [21] and CholecSeg8k [11]. To eliminate any potential data leakage, we exclude any images that appear in their validation or test sets from the pretraining dataset. Furthermore, all synthetic images are excluded. Outside the previously mentioned exceptions, we use all of the images from the nine datasets. For consistency, we always downsample to 1 FPS.

**Table 1:** An overview of the individual datasets that form the Endo700k dataset

Dataset	Surgery type	# Surg.	# Unique images
ESAD [3]	Robot-assisted radical prostatectomy	4	49,544
LapGyn4 (v1.2) [15]	Gynecologic laparoscopy	>500	38,192
Surgical Actions160 [23]	Gynecologic laparoscopy	59	761
GLENDA (v1.0) [14]	Gynecologic laparoscopy	>400	1,083
hSDB instrument [30]	Laparoscopic cholecystectomy	24	35,576
	Robotic gastrectomy	24	
HeiCo [18]	Laparoscopic proctocolectomy	10	347,257
	Laparoscopic rectal resection	10	
	Laparoscopic sigmoid resection	10	
PSI-AVA [27]	Robot-assisted radical prostatectomy	8	73,618
DSAD [4]	Robot-assisted rectal resection	32	13,195
Cholec80 [26]	Laparoscopic cholecystectomy	80	184,498
CholecT45 [21]	Laparoscopic cholecystectomy	45	0
CholecSeg8k [11]	Laparoscopic cholecystectomy	17	0

The first nine datasets (ESAD—Cholec80) represent a unique collection of roughly 744k raw endoscopic images. Cholec80 and its subvariants CholecT45 and CholecSeg8k are additionally used for downstream tasks of surgical phase recognition, action triplet recognition, and semantic segmentation

**Fig. 1:**
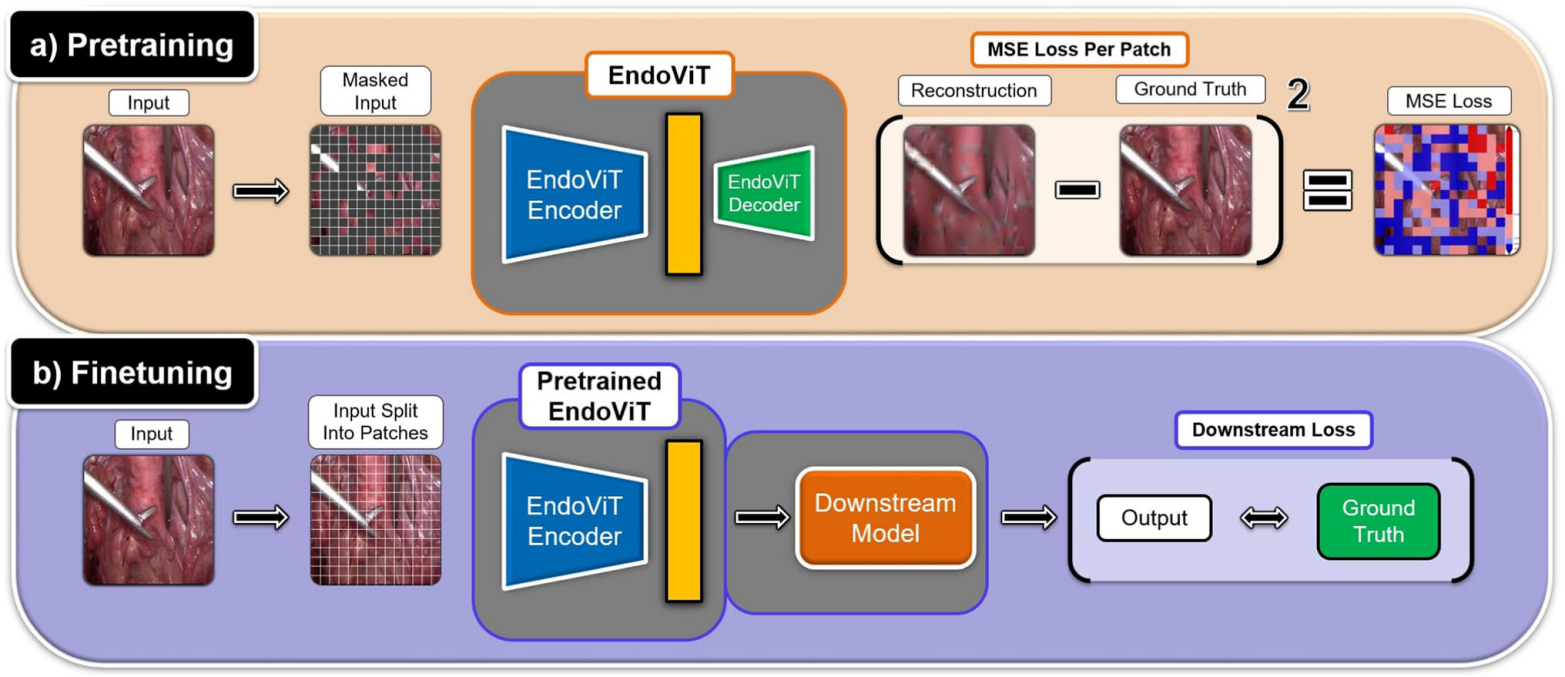
EndoViT is first pretrained using the Masked Image Modeling strategy (**a**). An input image is split into non-overlapping patches, and a large proportion of them is masked out. The network is trained to reconstruct the missing patches using a per-patch MSE loss, gaining a general visual understanding. Later, the EndoViT encoder can be finetuned and used as a powerful feature extraction backbone on downstream tasks (**b**). No Masking is applied during use as a feature extractor

### Model pretraining

Most existing works  [5, 6, 13, 21, 26, 27] use ImageNet-pretrained CNN models as image feature extraction backbones. However, ImageNet [7] contains natural images that differ significantly from endoscopic images. Therefore, in this work, we use Endo700k to pretrain a large-scale vision transformer-based [8] feature extractor on the endoscopy domain. The goal of the pretraining is to give a general understanding of the domain of endoscopic procedures to benefit a wide range of downstream tasks. For pretraining, we closely follow the approach of MAE [9] and employ the Masked Image Modeling strategy. The input image is first split into non-overlapping patches. Afterward, a large proportion of them is masked out. The network is trained to reconstruct the missing parts of the input. The encoder of the pretrained model can then be used as a feature extraction backbone in the downstream tasks. An overview of the pretraining procedure can be seen in Fig. 1. We tailor the MAE approach for the endoscopic setting with three modifications:

**Layerwise learning rate decay** We scale down the learning rate of each layer of the MAE encoder and decoder such that the layers closer to the latent space have larger learning rates, while those closer to the ends of the model have lower learning rates.

**Stochastic weight averaging (SWA) **[12]: During the last 5 pretraining epochs, we average the models’ weights at each validation step.

**Frequent evaluation** The evaluation is performed 6 times per epoch, and the best SWA model is saved.

#### Implementation details

We follow most of the practices and hyperparameter choices of [1, 9]. During pretraining only simple image augmentations are applied, including random resized crops and random horizontal flips. We use AdamW optimizer [16] with a learning rate of 1.5e$-$3 and batch size of 256. We pretrain for a total of 15 epochs. The training starts with 3 linear warmup epochs, continues according to the cosine scheduler until epoch 10, and ends with a constant learning rate applied during SWA. We use layer-wise learning rate decay of 0.65. Mean-squared error (MSE) is used as the reconstruction loss. We pretrain three different models, one for each of the downstream tasks. All are pretrained on Endo700k; however, the pretraining datasets are slightly different, obtained by removing validation and test datasets of CholecT45, Cholec80, and CholecSeg8k, respectively. All models have been implemented in PyTorch 1.13.0 and trained on 1 Nvidia a40 GPU.

**Table 2:** Semantic segmentation results, few shot, and full dataset (mean IoU)

	Train Set	ViT w/o Pretr	ViT ImageNet	EndoViT
Low Res	1 Video	29.11 ± 2.94	38.35 ± 8.27	**40.95 ± 10.32**
	2 Videos	36.28 ± 5.06	50.36 ± 2.71	**54.02 ± 4.18**
	4 Videos	43.29 ± 0.96	54.17 ± 2.35	**57.87 ± 2.70**
	Full	51.70 ± 0.54	62.45 ± 0.90	**65.05 ± 0.67**
High Res	1 Video	26.66 ± 6.64	39.06 ± 5.17	**41.16 ± 10.75**
	2 Videos	35.69 ± 4.45	50.14 ± 4.48	**56.05 ± 5.73**
	4 Videos	44.16 ± 0.75	56.22 ± 1.52	**59.81 ± 3.27**
	Full	53.18 ± 1.20	63.40 ± 0.81	**65.32 ± 0.56**

Bold values represent the best result

### Downstream tasks

After pretraining our feature extractor backbones, we evaluate their performance on three downstream tasks, namely semantic segmentation, action triplet recognition, and surgical phase recognition.

**Semantic segmentation** We choose the Dense Prediction Transformer (DPT) [22] architecture to leverage our vision transformer backbone for the semantic segmentation task. DPT assembles tokens from various stages of the ViT into image-like representations at various resolutions and progressively combines them [22]. Since the ViT backbone processes the input at high resolution and has a global receptive field, DPT allows for fine-grained and more globally consistent predictions compared to previous CNN approaches, especially when a larger amount of data can be provided [22]. We replace the encoder of DPT with our ViT encoder but otherwise keep the training setup the same. The DPT decoder is randomly initialized. We replicate the evaluation setup of [24].

**Fig. 2:**
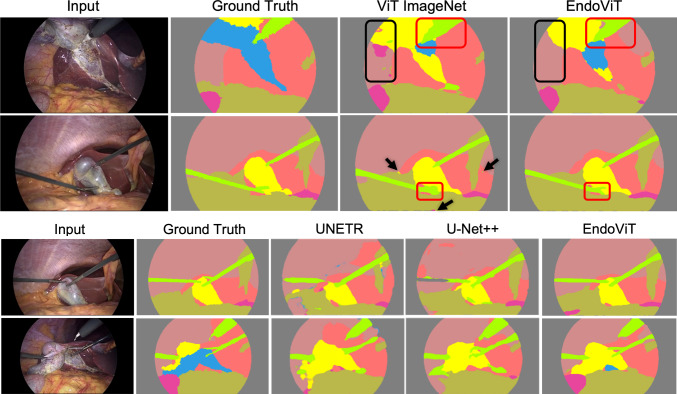
Qualitative segmentation comparison. EndoViT has more globally consistent outputs (highlighted in black) and is significantly better at reconstructing instrument tips (highlighted in red)

**Action triplet recognition** We build a straightforward model consisting of a feature extraction backbone and a linear head to detect the $<instrument, verb, target>$ triplets. To avoid data leakage into the pretraining set, we evaluate only on fold 5 as defined by [20]. We chose fold 5 specifically, as it yields the most balanced distribution of classes across train, val, and test splits, in the otherwise highly imbalanced CholecT45 dataset. While most works such as [19, 21] utilize Binary Cross-Entropy loss, we empirically find that Focal Loss brings significant improvement and therefore use it in all our experiments.

**Surgical phase recognition** In the task of Surgical Phase Recognition, the objective is to detect different phases of a surgical procedure based on the surgical video stream. For this task, we choose TeCNO [6], a well-known benchmark model with publicly available code. TeCNO is a two-step surgical phase recognition method. In the first step, a single-frame ResNet50 model is trained to predict surgical phases. In the second step, a Multi-Stage Temporal Convolutional Network (MS-TCN) refines the extracted features using temporal context. This two-stage approach allows the MS-TCN to improve the predictions of any feature extractor regardless of the chosen architecture. In our experiments, we replace the Resnet50 backbone with a ViT model and otherwise stick to the training and evaluation setup of TeCNO.

## Experiments

We compare our EndoViT (endoscopy pretrained) with its ImageNet pretrained ViT counterpart and commonly used CNN architectures (ResNet50/ResNet18 [10]). We evaluate the performance of the models on the full downstream dataset and also evaluate the few-shot learning performance using a reduced amount of training videos while keeping the validation and test sets the same. We report the mean and standard deviation of the corresponding metrics across 3 runs for each network in each setting.

**Semantic segmentation** We report the semantic segmentation results in Table 2. The reported metric is mean Intersection over Union (IoU). The results are reported both for EndoViT’s pretraining resolution of 224*x*224 (Low Res) and the resolution used in [24] of 256*x*448 (High Res). EndoViT outperforms the ImageNet pretrained ViT in all scenarios, including few shot, with a margin of 2–6%. We report a comparison to other methods from [24] in Fig. 2 and Table 3. EndoViT outperforms other Transformers (UNETR) as well as various CNN architectures, including U-Net++, the previous SOTA to our knowledge, by a significant margin of 10%. EndoViT shows more globally consistent predictions and is better at reconstructing the crucial instrument tips.

**Action triplet recognition** We report the results on the full dataset in Table 4. The reported metric is mean Average Precision (mAP) proposed by the authors of the CholecT45 dataset [21]. The results show that EndoViT outperforms both CNN architectures and its ImageNet pretrained counterpart by 8% and 2%, respectively, empirically showcasing the value of using endoscopy-based models. Furthermore, from the performance of the randomly initialized ViT model, it can be seen that pretraining is essential for vision transformers. In Table 5, we report few-shot learning experiment results by training only on 2, 4, or 8 videos. We observe the same trends in our few-shot learning experiments. EndoViT outperforms ResNet50 by 5–6.5% and the ImageNet model by 2–5%.

**Table 3:** Semantic segmentation comparison to previous methods (mean IoU)

U-Net++	DynUNet	UNETR	DeepLab V3+	EndoViT
55	52	49	50	**65.32 ± 0.56**

Bold values represent the best result

Results for methods other than ours from [24]

**Table 4:** Action triplet recognition full dataset results (mAP)

ResNet18	ResNet50	ViT w/o Pretr	ViT ImageNet	EndoViT
21.72 ± 1.17	22.13 ± 1.37	13.93 ± 0.43	27.84 ± 0.39	**30.17 ± 0.01**

Bold values represent the best result

**Surgical phase recognition** We report the results on the full dataset in Table 6. The reported metric is Accuracy averaged over all testing videos. We report the performance of all models after both stages of TeCNO training. For reference purposes, we note that the reported performance of ResNet50 backbone in TeCNO [6] is 88.56% ± 0.27%. Once again, the CNN architecture is outperformed by the pretrained vision transformers. However, the difference between EndoViT and ImageNet pretrained backbone is negligible in both stages. We believe there are two causes. One, the semantic understanding induced by reconstructing image patches is not capable of capturing the long-term relationships required to discriminate between different surgical phases accurately. And two, the training set used in Cholec80 is relatively large (approx. 90k images), making it easier to overcome the pretraining differences. In Table 7, we report few-shot learning experiment results by training only on 2, 4, or 8 videos. ResNet50 showcases a significant decrease in performance. When training on 2 videos only, EndoViT outperforms its ImageNet counterpart in both stages. For 4 and 8 videos, we observe comparable performance.

**Table 5:** Action triplet recognition few-shot results (mAP)

	ResNet50	ViT ImageNet	EndoViT
2 Videos	10.88 ± 0.50	12.22 ± 1.78	**17.59 ± 2.94**
4 Videos	12.37 ± 1.78	14.27 ± 1.73	**18.52 ± 2.28**
8 Videos	17.01 ± 1.75	19.71 ± 0.61	**21.91 ± 0.12**

Bold values represent the best result

**Table 6:** Surgical phase recognition full dataset results (mean Accuracy)

	ResNet50	ViT w/o Pretr	ViT ImageNet	EndoViT
Stage 1	79.84 ± 0.30	59.21 ± 0.36	**82.94 ± 0.69**	82.60 ± 1.26
Stage 2	87.84 ± 0.58	73.42 ± 0.70	**89.56 ± 0.65**	89.37 ± 0.95

Bold values represent the best result

**Table 7:** Surgical phase recognition few-shot results (mean accuracy)

	Train Set	ResNet50	ViT ImageNet	EndoViT
Stage 1	2 Videos	47.51 ± 1.33	63.59 ± 1.07	**67.04 ± 2.92**
	4 Videos	57.80 ± 2.67	67.72 ± 0.90	**71.80 ± 0.49**
	8 Videos	63.71 ± 1.48	**75.50 ± 0.32**	75.30 ± 1.83
Stage 2	2 Videos	68.23 ± 1.10	77.05 ± 1.71	**78.89 ± 1.26**
	4 Videos	74.50 ± 1.76	80.00 ± 0.62	**80.28 ± 0.71**
	8 Videos	77.43 ± 1.68	84.10 ± 0.38	**84.68 ± 1.25**

## Conclusion

EndoViT performs equally or better than the same ViT model pretrained on ImageNet in all of our experiments. We observe that the improvements from our endoscopy-pretraining were more pronounced in the more complex downstream tasks. In action triplet recognition, endoscopy-specific pretraining significantly outperforms ImageNet pretraining. For the simpler task of surgical phase recognition, our pretraining was less impactful, although never hurting performance. In the most complex task of semantic segmentation, our EndoViT model outperforms the previous SOTA. Moreover, our method performing well on all of the diverse downstream tasks shows that our pretraining implementation, which reconstructs image patches in pixel space, captures general information about the objects and scenes it has seen. We therefore conclude that EndoViT would be an excellent upgrade to the ImageNet pretrained feature extraction backbones that many surgical video understanding methods rely on. We hope that the release of our dataset collection Endo700k, pretraining implementation, and pretrained EndoViT model will help the community to solve more challenging tasks with the small amount of data available.
